# Amalgam Phase-Down Part 2: UK-Based Knowledge, Opinions, and Confidence in the Alternatives

**DOI:** 10.1177/2380084420954766

**Published:** 2020-12-10

**Authors:** O. Bailey, C.R. Vernazza, S. Stone, L. Ternent, A.-G. Roche, C. Lynch

**Affiliations:** 1 School of Dental Sciences, Newcastle University, Newcastle upon Tyne, UK; 2 Population Health Sciences Institute, Newcastle University, Newcastle upon Tyne, UK; 3 British Dental Association, London, UK; 4 University Dental School & Hospital, University College Cork, Cork, Ireland

**Keywords:** caries treatment, health services research, restorative dentistry, restorative materials, composite materials, clinical outcomes

## Abstract

**Introduction:**

Amalgam use has recently been phased down, and the potential for a phase-out is being investigated.

**Objectives:**

The study aimed to identify knowledge of the phase-down and opinions of a potential phase-out of amalgam by UK primary care clinicians and assess their confidence in using different materials in different situations.

**Methods:**

An anonymized, prepiloted cross-sectional e-survey was used to assess primary care clinicians’ knowledge and opinions of the amalgam phase-down and potential phase-out and their confidence in using amalgam and the alternatives in different situations. In total, 11,902 invitations were distributed through British dentist and therapist associations. Prior hypotheses were tested alongside descriptive statistics.

**Results:**

Response rate was 13% (*n* = 1,513). Knowledge of the amalgam phase-down was low, with just 3% clinicians correctly identifying all patient groups in whom amalgam use should be avoided in the United Kingdom. Postgraduate education on posterior composite placement was high (88%), but a large majority had personal and patient-centered concerns over the suitability of the alternatives and lacked confidence when placing composite in comparison to amalgam in difficult situations (*P* < 0.0001). Logistic regressions revealed that the best predictors of high confidence in placing mesio-occluso-distal composites and composites in difficult situations were being a private general dentist or being primarily a composite user.

**Conclusion:**

Primary care clinicians have major personal and patient-centered concerns regarding the amalgam phase-down (of which they have limited knowledge) and potential phase-out. Many lack confidence in using the alternative, composite, to restore posterior teeth in difficult situations, whereas confidence in using amalgam in similar situations is high. Effective education of clinicians and understanding patients’ needs, alongside policy changes, are required to enable a successful amalgam phase-down and potential phase-out.

**Knowledge Transfer Statement:**

This study shows that UK primary care clinicians are worried about the phase-down of amalgam for themselves and their patients. Many lack confidence in the alternative, composite, when used in difficult situations, which is in stark contrast to amalgam. Knowledge of the phase-down is limited. There is a need for more effective education of clinicians, an understanding of patients’ values, and policy changes to ensure the success of the phase-down and potential phase-out of amalgam.

## Introduction

This is the second of 2 articles detailing a UK survey of primary care dentists and therapists exploring opinions, techniques, and materials used for the provision of direct posterior restorations. This article focuses on clinicians’ opinions and knowledge of the phase-down and potential phase-out of amalgam while assessing their confidence in using different materials in different situations.

The Minamata Convention on Mercury was agreed on in 2013, prescribing an amalgam phase-down to protect the environment (Minamata Convention on Mercury 2013). This has been implemented by the European Parliament, which introduced a phase-down in July 2018 while also stating that the feasibility of a phase-out by 2030 should be investigated (Regulation (EU) 2017/852 2017).

Evidence exists from around the world on dentists’ opinions of an amalgam phase-down (Alexander et al. 2014; Callanan et al. 2020) and phase-out from countries where amalgam has been banned (Kopperud et al. 2016). The cost of the amalgam phase-out in Norway, for example, has mostly been borne by patients and providers, and the use of amalgam prior to the phase-out was low (Norwegian Climate and Pollution Agency 2012). The context of health care provision is very different from that of the United Kingdom, however, where publicly funded National Health Service (NHS) with some copayment provision predominates, with amalgam still being commonly used (Lynch et al. 2018).

A recent study provided data on the opinions of NHS general dentists (GDs) on the phase-down and potential phase-out of amalgam limited to Wales (Lynch et al. 2018). While confidence in placing composite in different situations was assessed, confidence in placing amalgam was not assessed, making the potential impact of a phase-out difficult to quantify. A large majority did not feel confident in placing direct posterior composites in cavities with subgingival margins, which is a concern, but it was unclear if this was also an issue when using amalgam. However attendance on postgraduate courses on posterior composites was also low (16% of respondents) (Lynch et al. 2018). Opinions were not sought from community dental service (CDS) dentists, who work with more challenging patients (e.g., those with special requirements or behavioral issues) and worry that the amalgam phase-out could widen already existing health inequalities (Steele et al. 2015; M. West, personal communication, 2018), or the growing UK therapist workforce (Centre for Workforce Intelligence 2014), making the potential impact of the phase-down on primary care difficult to assess.

A majority also felt there was an issue of longevity with composite compared to amalgam (Lynch et al. 2018). This is supported by stringently assessed clinical trial data (Rasines Alcaraz et al. 2014; Khangura et al. 2018), but the discrepancy is not as great as seen in the practice environment, both in the United Kingdom and Scandinavia, where cross-sectional data suggest a greater disparity (Burke et al. 1999; Forss and Widström 2001; Sunnegardh-Gronberg et al. 2009), which is clearly of concern for both tooth survival and the likely lifetime costs of replacement.

Given that an amalgam phase-down has recently been implemented in the United Kingdom, which is still an area of high amalgam use, the objectives of this study were to determine different primary care clinicians’ knowledge of newly imposed restrictions, opinions on the phase-down and potential phase-out (including confidence in placement of the available direct posterior restorative materials in various situations), and educational experience related to posterior composites while determining differences between subgroups.

## Methods

An anonymized cross-sectional e-survey (available on request from the authors) was developed to assess clinicians’ opinions and confidence in amalgam and the alternatives in various situations, as well as knowledge of the amalgam phase-down and proposed phase-out. Clinicians’ experience of undergraduate and postgraduate education on direct posterior composites was also assessed. The questionnaire used Likert instruments and open and closed questions based on previous studies (Alexander et al. 2016; Lynch et al. 2018), which were modified in relation to best practice methodology (Dillman et al. 2014) and prepiloting to minimize survey error. Strengthening the Reporting of Observational Studies in Epidemiology (STROBE) guidelines were followed and a favorable ethical opinion was obtained from the Newcastle University Research Ethics Committee (ref. 7262/2018).

Further details of the methods used have been described elsewhere (Bailey et al. 2022).

### Sample

A sample size calculation based on core analysis has previously been described, obtaining an estimate of 630 (Bailey et al. 2022). The questionnaire underwent email distribution on February 14, 2019, to all therapist members of the British Association of Dental Therapists (BADT) and the British Society of Dental Hygiene and Therapy (BSDHT), as well as all GD and CDS members of the British Dental Association (BDA) (11,902 invitations), with a deadline for response March 31, 2019. The therapist sampling frame was open with no incentivization, whereas the dentist sampling frame was closed with a random draw £100 incentive provided for 1 respondent. Two reminders were sent. Eligibility, understanding, and consent for participation were confirmed with yes/no questions. Data were automatically electronically captured by the BDA and passed securely to Newcastle University for analysis.

### Data Analysis

Stata software (version 16; StataCorp LP) was used to import, clean, and analyze the data. Basic statistical testing was performed. Wilcoxon signed rank tests were used to analyze differences in confidence in placing direct posterior restorations, of composite or amalgam, with subgingival margins, and in patients with limited cooperation. Differences in response between clinicians relating to knowledge of the amalgam phase-down were analyzed using χ^2^ tests. Clinician and technique-based factors associated with high or complete confidence in placing direct posterior composite restorations in various situations (mesio-occluso-distal [MOD] cavity, subgingival margins, and in patients with limited cooperation) were analyzed using logistic regressions (using backward stepwise elimination). Lowest Bayesian information criterion values were used to select the models of best fit. Variance inflation factors were calculated to assess multicollinearity, with all values lower than 2.5. Data, samples, or models will be provided on request to the corresponding author.

## Results

Of the 1,570 responses received, 1,513 were usable. Fifty-four respondents were not suitable to participate, and 3 respondents were but failed to answer any questions. The response rate was 14% for dentists and an estimated minimum of 6% for therapists. Survey completion rate was 99.8% for eligible responders.

Direct posterior restorations throughout this report exclude localized cervical (class V) restorations, and percentages are rounded to the nearest integer. Demographic data have already been presented (Bailey et al. 2022), but there was good representation of groups by sex, years qualified, and practicing arrangement. Given that dental workforce demographics are not published, it is not possible to judge how representative the sample is.

### Education in Direct Posterior Composite

As undergraduates, 30% respondents had not received didactic teaching and 36% had not received clinical teaching on direct posterior composites, with 7% unable to remember. A high proportion of respondents had attended a postgraduate course on direct posterior composite placement (88%) (Appendix Table 1).

**Table 1.** Opinions Relating to the Potential Phase-out of Amalgam.

Opinion Relating to the Phase-out of Amalgam	Agree or Strongly Agree (%)	Neither Agree nor Disagree (%)	Disagree or Strongly Disagree (%)
Will impact on my ability to do my job (*n* = 1,506)	65	12	23
Will lead to the need for more indirect restorations (*n* = 1,508)	71	14	15
Will lead to more teeth being deemed unrestorable (*n* = 1503)	62	14	25
There is a lack of consensus on best practice when selecting direct alternative materials (*n* = 1,506)	69	19	12
There is a lack of consensus on best practice in terms of technique when directly placing alternative materials (*n* = 1,503)	61	22	17
My patients won’t care (*n* = 1,506)	23	27	50
Suitable directly placed alternatives to amalgam are available (*n* = 1,497)	45	14	41
I feel up to date with current techniques and practices relating to placement of posterior composites (*n* = 1,495)	76	14	10
Having to routinely place posterior composites would cause appointment delays in my practice (*n* = 1,493)	62	11	27
Posterior amalgams last longer than directly placed posterior composites (*n* = 1,498)	62	24	14
It takes me longer to remove a failed posterior composite restoration than a failed amalgam restoration of equivalent size (*n* = 1,498)	70	14	16

### Amalgam Phase-Down and Proposed Phase-Out

Respondents’ knowledge of the amalgam phase-down was ascertained by asking them to state in which patient groups amalgam use should currently be avoided (Appendix Table 2) and by which year the phase-out was planned.

**Table 2.** Clinician Confidence in Providing Various Restorations in Varying Clinical Situations.

Clinician Confidence Level	No or Low Confidence (%)	Moderate Confidence (%)	High or Complete Confidence (%)
In providing 2 surface direct posterior composite restorations involving a proximal surface (*n* = 1,507)	2	19	79
In providing 3 surface direct posterior composite restorations involving both proximal surfaces (*n* = 1,501)	5	27	67
In providing definitive 2 surface posterior GIC restorations involving a proximal surface (*n* = 1,503)	23	31	45
In providing definitive 3-surface posterior GIC restorations involving both proximal surfaces (*n* = 1,501)	31	30	39
When placing direct posterior composites with subgingival margins (*n* = 1,505)	51	31	18
When placing posterior amalgams with subgingival margins (*n* = 1,476)	4	18	78
When placing direct posterior composites in patients with limited cooperation (*n* = 1,505)	69	23	8
When placing posterior amalgams in patients with limited cooperation (*n* = 1,483)	7	46	48

GIC, glass ionomer cement.

Forty percent (40%) respondents correctly identified the year (2030) of the proposed phase-out of amalgam (dentists 40%, therapists 38%; no statistically significant difference between groups, χ^2^ *P* = 0.701) (*n* = 1,481). Fifty-one percent thought it was prior to this. Only 3% of dentists and therapists correctly identified all patient groups in which the use of amalgam should be avoided according to current rules (Regulation (EU) 2017/852 2017). There was no statistically significant difference between the clinicians (χ^2^ *P* = 0.883).

Clinicians were also asked their opinions about various aspects of the phase-down based on a 5-point Likert scale. Responses for *strongly agree* and *agree*, as well as *strongly disagree* and *disagree*, were combined and are presented in Table 1. A large majority felt that the phasing out of amalgam would affect their ability to do their job and lead to the need for more indirect restorations and more teeth being deemed unrestorable, and they also believed that there is a lack of consensus on best practice in both material selection and technique when placing alternatives to amalgam but felt up to date with current techniques and practices relating to placement of direct posterior composite restorations. A majority felt that their patients would care about the phasing out of amalgam, and a large majority felt that posterior amalgams last longer than posterior composite restorations, that having to routinely place posterior composite restorations would lead to appointment delays in their practice, and that it takes longer to remove a failed posterior composite than a failed amalgam restoration of equivalent size.

Clinicians were asked over which period of time they felt amalgam should be phased out from UK dental practice. The responses (*n* = 1494) were as follows: less than 5 y, 21%; 5 to 9 y, 23%; 10 to 19 y, 24%; 20 to 29 y, 7%; and ≥30 y, 26%.

### Clinician Confidence

Clinicians were asked to state how confident they were placing direct posterior restorations in different clinical situations based on a 5-point Likert scale. Responses for “complete confidence” and “high confidence,” as well as “no confidence” and “low confidence,” were combined and are presented in Table 2.

Wilcoxon signed rank tests showed statistically significantly lower (*P* < 0.0001) clinician confidence when placing direct posterior restorations with subgingival margins, as well as in patients with limited cooperation, when using composite compared to amalgam. The difference was marked, with 51% reporting no or low confidence when placing a direct posterior composite with subgingival margins, compared to just 4% when placing amalgam in the same situation, and 69% reporting no or low confidence when placing a direct posterior composite in patients with limited cooperation, compared to just 7% when placing amalgam in the same situation. Clinicians generally had high or complete confidence in placing direct posterior composites involving both proximal surfaces.

#### Regression analyses

Pseudo-*R*^2^ values suggested the models explained only a small portion of the variance for all of the regression analyses performed. The significant factors in each model are discussed below, however.

Table 3 details the logistic regression to explore the influence of various factors on confidence in placing direct posterior MOD composite restorations.

**Table 3.** Factors Related to High or Complete Confidence in Placing Direct Posterior Mesio-Occluso-Distal Composite Restorations: A Logistic Regression Analysis.

Independent Variable (Predictor)	Odds Ratio	Standard Error	*z*	*P* > *z*	95% Confidence Interval
No undergraduate clinical teaching (reference had UG teaching)	0.57	0.13	−2.48	**0.013**	0.37–0.89
No postgraduate training (reference had PG training)	0.81	0.22	−0.74	0.457	0.48–1.40
UK primary dental qualification (reference non-UK)	0.67	0.21	−1.27	0.204	0.37–1.24
Type of practice (reference NHS general dentist 75%–100% NHS patient base)					
Private general dentist (0%–24% NHS patient base)	2.20	0.62	2.80	**0.005**	1.27–3.81
Mixed general dentist (25%–74% NHS patient base)	2.13	0.63	2.58	**0.010**	1.20–3.79
CDS dentist	0.37	0.13	−2.80	**0.005**	0.18–0.74
Therapist	0.34	0.16	−2.37	**0.018**	0.14–0.83
Years qualified	1.00	0.01	0.23	0.816	0.98–1.02
Female (reference male)	0.64	0.13	−2.27	**0.023**	0.44–0.94
Composite user (combined premolar and molar composite usage >100%) (reference combined use <100%)	2.02	0.46	3.07	**0.002**	1.29–3.17
Incremental composite user (76%–100% use) (reference <76% incremental)	1.09	0.21	0.45	0.653	0.75–1.59
Bonding system use (reference self-etch 1 step [76%−100% use])					
Total-etch 3-step bond (76%−100% use)	1.31	0.50	0.70	0.485	0.62−2.77
Total-etch 2-step bond (76%−100% use)	1.08	0.28	0.28	0.781	0.65−1.79
Self-etch 2-step bond (76%−100% use)	0.98	0.75	−0.02	0.984	0.22−4.39
Matrix use (reference not CM or SM user)					
Circumferential metal user (100% use)	1.69	0.34	2.61	**0.009**	1.14–2.50
Sectional metal user (51%−100% use)	1.73	0.54	1.78	0.075	0.95−3.18
High wedge use (76%−100% use) (reference <76% use)	1.10	0.22	0.50	0.616	0.75−1.62
Never liner use (reference >0% use)	1.30	0.28	1.21	0.225	0.85−1.97
Rubber dam use (reference 1%−75% use)					
Never	0.93	0.19	−0.37	0.712	0.61−1.40
High (76%–100% use)	1.072	0.35	0.21	0.833	0.56−2.05
Agree lack of consensus on material (reference don’t agree)	0.75	0.21	−1.05	0.292	0.43–1.30
Agree lack of consensus on technique (reference don’t agree)	0.56	0.14	−2.38	**0.017**	0.34–0.90
Disagree suitable alternatives to amalgam exist (reference don’t disagree)	0.69	0.13	−1.97	**0.049**	0.48–1.00
Low reported sensitivity (0%−10%) (reference ≥11% sensitivity)	1.34	0.27	1.43	0.153	0.90−2.00
Low reported food packing (0%−10%) (reference ≥11% FP)	2.13	0.43	3.75	**0.000**	1.44−3.17
Constant	2.14	1.11	1.47	0.142	0.77

*n* = 768; *P* < 0.0001; pseudo-*R*^2^ = 0.22.

CDS, community dental service; CM, circumferential matrix; FP, food packing; NHS, National Health Service; PG, postgraduate; SM, sectional matrix; UG, undergraduate.

Type of practice significantly affected confidence in placing a direct posterior MOD composite, with private GDs and mixed GDs more than twice as likely to be confident compared to NHS GDs, whereas CDS dentists and therapists were less than half as likely to be confident. Primarily composite users and clinicians reporting a low incidence of postoperative food packing after composite placement were twice as likely to be confident, with those using circumferential metal matrices 1.7 times as likely to be confident in placing direct posterior MOD composites. Clinicians who were female (odds ratio [OR] = 0.6), those who agreed that there was a lack of consensus on composite technique (OR = 0.6), and those who disagreed (or strongly disagreed) that suitable alternative to amalgam existed (OR = 0.7) were less likely to be confident in placing direct posterior MOD composite restorations.

Table 4 details the regression to explore the influence of various factors on confidence in placing direct posterior composites with subgingival margins.

**Table 4.** Factors Related to High or Complete Confidence When Placing Direct Posterior Composites with Subgingival Margins: A Logistic Regression Analysis.

Independent Variable (Predictor)	Odds Ratio	Standard Error	*z*	*P* > *z*	95% Confidence Interval
No undergraduate clinical teaching (reference had UG teaching)	0.67	0.18	−1.52	0.129	0.40−1.12
No postgraduate training (reference had PG training)	1.07	0.43	0.16	0.876	0.48–2.35
UK primary dental qualification (reference non-UK)	0.47	0.14	−2.45	**0.014**	0.26–0.86
Type of practice (reference NHS general dentist [75%–100% NHS patient base])					
Private general dentist (0%−24% NHS patient base)	2.47	0.80	2.81	**0.005**	1.31–4.65
Mixed general dentist (25%−74% NHS patient base)	1.66	0.60	1.41	0.158	0.82−3.36
CDS dentist	0.61	0.41	−0.73	0.466	0.17–2.28
Therapist	1.04	0.70	0.06	0.953	0.28–3.91
Years qualified	0.99	0.01	−0.64	0.520	0.97–1.02
Female (reference male)	0.58	0.13	−2.34	**0.019**	0.37–0.92
Composite user (combined premolar and molar composite usage >100%) (reference combined use <100%)	1.83	0.51	2.17	**0.030**	1.06–3.15
Incremental composite user (76%−100% use) (reference <76% incremental)	1.18	0.26	0.76	0.446	0.77−1.82
Bonding system use (reference self-etch 1 step [76%–100% use])					
Total-etch 3-step bond (76%−100% use)	0.65	0.25	−1.13	0.257	0.31−1.37
Total-etch 2-step bond (76%−100% use)	0.64	0.17	−1.70	0.089	0.38–1.07
Self-etch 2-step bond (76%−100% use)	0.83	0.57	−0.27	0.789	0.22–3.18
Matrix use (reference not CM or SM user)					
Circumferential metal user (100% use)	1.05	0.27	0.18	0.856	0.64−1.73
Sectional metal user (51%−100% use)	0.96	0.28	−0.13	0.900	0.55−1.70
High wedge use (76%−100% use) (reference <76% use)	0.62	0.15	−1.92	0.055	0.38−1.01
Never liner use (reference >0% use)	1.36	0.30	1.37	0.171	0.88−2.11
Rubber dam use (reference 1%−75% use)					
Never	0.98	0.26	−0.07	0.941	0.58–1.65
High (76%–100% use)	2.17	0.65	2.56	**0.010**	1.20–3.92
Agree lack of consensus on material (reference don’t agree)	0.80	0.22	−0.80	0.425	0.46–1.39
Agree lack of consensus on technique (reference don’t agree)	0.75	0.20	−1.05	0.293	0.44–1.28
Disagree suitable alternatives to amalgam exist (reference don’t disagree)	0.59	0.14	−2.19	**0.029**	0.36–0.95
Low reported sensitivity (0%–10%) (reference ≥11% sensitivity)	0.77	0.20	−1.00	0.316	0.47–1.28
Low reported food packing (0%–10%) (reference ≥11% FP)	2.59	0.70	3.51	**0.000**	1.52–4.41
Constant	0.42	0.24	−1.55	0.122	0.14–1.26

*n* = 768; *P* < 0.0001; pseudo-*R*^2^ = 0.17.

CDS, community dental service; CM, circumferential matrix; FP, food packing; NHS, National Health Service; PG, postgraduate; SM, sectional matrix; UG, undergraduate.

Private GDs were 2.5 times as likely to be confident in placing composites with subgingival margins compared to NHS GDs. Clinicians whose patients reported low postoperative food packing following direct posterior composite placement were 2.6 times as likely to be confident, those with high rubber dam use over twice as likely to be confident, and those primarily using composite 1.8 times as likely to be confident. Those with a UK primary qualification were less than half as confident, and female clinicians and those who disagreed that suitable alternatives to amalgam existed were 0.6 times as confident in placing direct posterior composites with subgingival margins.

Table 5 details the regression to explore the influence of various factors on confidence in placing direct posterior composites in patients with poor cooperation.

**Table 5.** Factors Related to High or Complete Confidence in Placing Composites in Patients with Poor Cooperation: A Logistic Regression Analysis.

Independent Variable (Predictor)	Odds Ratio	Standard Error	*z*	*P* > *z*	95% Confidence Interval
No undergraduate clinical teaching (reference had UG teaching)	1.22	0.44	0.57	0.570	0.61–2.46
No postgraduate training (reference had PG training)	1.53	0.82	0.80	0.426	0.54–4.35
UK primary dental qualification (reference non-UK)	0.34	0.13	−2.80	**0.005**	0.16–0.73
Type of practice (reference NHS general dentist [75%−100% NHS patient base])					
Private general dentist (0%−24% NHS patient base)	2.69	1.26	2.11	**0.035**	1.07−6.74
Mixed general dentist (25%−74% NHS patient base)	2.63	1.34	1.90	0.057	0.97–7.14
CDS dentist	1.50	1.11	0.55	0.580	0.35–6.39
Therapist	3.05	2.29	1.49	0.137	0.70–13.27
Years qualified	0.98	0.02	−0.93	0.351	0.95–1.02
Female (reference male)	0.96	0.31	−0.12	0.905	0.52–1.79
Composite user (combined premolar and molar composite usage >100%) (reference combined use <100%)	2.00	0.79	1.77	0.077	0.93–4.32
Incremental composite user (76%–100% use) (reference <76% incremental)	1.27	0.39	0.79	0.431	0.70−2.32
Bonding system use (reference self-etch 1 step [76%−100% use])					
Total-etch 3-step bond (76%−100% use)	1.51	0.75	0.82	0.413	0.57–4.01
Total-etch 2-step bond (76%−100% use)	1.32	0.52	0.70	0.485	0.61–2.85
Self-etch 2-step bond (76%−100% use)	1	(omitted)			
Matrix use (reference not CM or SM user)					
Circumferential metal user (100% use)	1.56	0.54	1.27	0.203	0.79–3.08
Sectional metal user (51%−100% use)	1.12	0.45	0.27	0.786	0.50–2.48
High wedge use (76%−100% use) (reference <76% use)	0.49	0.17	−2.07	**0.038**	0.25–0.96
Never liner use (reference >0% use)	1.05	0.33	0.15	0.884	0.57–1.93
Rubber dam use (reference 1%−75% use)					
Never	0.65	0.25	−1.10	0.270	0.31–1.39
High (76%−100% use)	1.83	0.74	1.49	0.137	0.80–4.04
Agree lack of consensus on material (reference don’t agree)	0.52	0.20	−1.73	0.083	0.24–1.09
Agree lack of consensus on technique (reference don’t agree)	1.05	0.39	0.12	0.904	0.50–2.18
Disagree suitable alternatives to amalgam exist (reference don’t disagree)	0.38	0.14	−2.57	**0.010**	0.18–0.79
Low reported sensitivity (0%−10%) (reference ≥11% sensitivity)	1.55	0.58	1.19	0.236	0.75–3.21
Low reported food packing (0%–10%) (reference ≥11% FP)	1.49	0.57	1.05	0.292	0.71–3.15
Constant	0.09	0.07	−3.12	0.002	0.02–0.40

*n* = 768; *P* < 0.0001; pseudo-*R*^2^ = 0.17.

CDS, community dental service; CM, circumferential matrix; FP, food packing; NHS, National Health Service; PG, postgraduate; SM, sectional matrix; UG, undergraduate.

Private GDs were 2.7 times more likely to be confident in placing direct posterior composites in patients with poor cooperation than NHS GDs. Those with a UK primary qualification were only 0.3 times as confident, those who disagree that suitable alternatives to amalgam exist 0.4 times as confident, and those with high wedge use 0.5 times as confident in placing direct posterior composites in patients with poor cooperation.

## Discussion

This study aimed to explore different primary care clinicians’ opinions and knowledge related to the newly imposed amalgam phase-down and potential phase-out (including confidence in the various materials used for direct restoration of posterior teeth in various situations) and educational experience related to posterior composites.

Comprehensive knowledge of the phase-down and phase-out of amalgam is low among primary care clinicians, which is of concern given that phase-down regulations are currently in place. Members of the associations from which the sample was drawn might be expected to be more informed than nonmembers, given much information has been repeatedly disseminated by each association on this topic. It seems likely that some respondents looked up the guidelines on the Internet, seemingly quoting previous Norwegian guidelines (Norwegian Climate and Pollution Agency 2012), which are different from UK guidelines (Regulation (EU) 2017/852 2017). Alternative modes of dissemination should be explored.

A large majority felt concerned about the potential phasing out of amalgam, feeling that issues existed over the suitability of alternatives and that amalgam restorations last longer than composite restorations (62%). This is in agreement with the opinions of Welsh dentists (57%) (Lynch et al. 2018) and Norwegian dentists after the implementation of the amalgam ban (a clinical vignette showed a class 2 restoration requiring replacement, with 71% dentists indicating that an amalgam restoration would last longer than a composite) (Kopperud et al. 2016). Clinical data also support this perception, both trial based and, importantly for consideration of primary care, cross-sectional based, which show marked differences in survival between composite and amalgam (Burke et al. 1999; Forss and Widström 2001; Sunnegardh-Gronberg et al. 2009; Rasines Alcaraz et al. 2014; Khangura et al. 2018). Not all data reviews agree with this, but these are primarily based on short-term clinical trial data (Heintze and Rousson 2012), with included individual studies often excluding patients at higher risk of restoration failure, for example, those with high caries risk, poor oral hygiene, and bruxism (Gallo et al. 2005), or include extensive retrospective data specific to a single dental practice (Opdam et al. 2014), all of which make translation of the data to primary care difficult.

A high proportion of respondents had attended a postgraduate course on direct posterior composite placement (88%), which was much higher than another recent survey sampling dentists in Wales (16%) prior to the implementation of the phase-down (Lynch et al. 2018). While this is encouraging, it did not translate to higher confidence in placement of posterior composites among the respondents in comparison to the Welsh study, with proportionally fewer respondents confident in placing an MOD composite (67% vs. 88%). This could be partially explained by the Welsh data being at risk of acquiescence bias. However, when these data are combined with the fact that only a small minority felt confident in placing composites in difficult situations, for example, in teeth with subgingival margins, the efficacy of current postgraduate education courses must be questioned, given relatively simple techniques, usable by GDs, have been described to manage such situations (Bailey and O’Connor 2019). These data are in marked contrast to the high confidence of a large majority of respondents when placing amalgam in similar, difficult situations, which is therefore a concern in light of the amalgam phase-down.

With a large majority feeling a phase-out would affect their ability to do their job, concerned by the extra time it would take to place and replace alternatives (supported by experimental data; Krejci et al. 1995), the consequent appointment delays, the increased need for indirect restorations and that more teeth would be deemed unrestorable, the potential impact on health care accessibility, cost, tooth loss, patient safety, dentist well-being, and the already widening oral health inequalities (Steele et al. 2015) is worrying. Respondents generally also felt that their patients would be concerned about a potential phase-out of amalgam (50%), which is very different from data collected from dentists in Australia, where amalgam use is low, with only 16% feeling similarly (Alexander et al. 2016). This is likely primarily due to the difference in public versus private service provision between the countries.

UK graduates were much less confident in placing composites in difficult situations than those qualifying from the rest of the world, which raises questions over UK education and the predominance and impact of publicly funded practicing arrangements, which favor amalgam placement in the United Kingdom (Lynch et al. 2018).

Primarily being a composite placer is a good predictor for high confidence in placing MOD composites and placing composites with subgingival margins. The practicing arrangement in the UK potentially limits clinician skill development, as is required for placing posterior composite restorations compared with amalgam (Kielbassa et al. 2016) and therefore confidence. This affects patient outcomes, as supported by data showing that primarily being a composite placer was the best predictor for low reported postoperative incidence of complications when placing direct posterior composites (Bailey et al. 2022). This would support the notion that repeatedly using a skill engenders competence and confidence, but repetition per se and confidence do not necessarily reflect competence (Morgan and Cleave-Hogg 2002; Davis et al. 2006). Evidence suggests that repetition of a skill needs to be deliberate and focused following insightful reflection for improvement to occur (Ericsson and Pool 2016). The nature of the patient population seen in the different sectors may differ, in terms of disease prevalence and extent, or compliance, for example, with NHS GDs potentially seeing more challenging patients in this regard than private GDs. This may also explain some of the differences seen in confidence between the practitioner groups.

CDS dentists tend to face more challenging patients, often with limited cooperation (M. West, personal communication, 2018), which makes composite placement more difficult due to the material’s technique sensitivity, which could account for their lower likelihood of confidence. The therapist cohort reported very high levels of postoperative sensitivity following the placement of composite restorations (Bailey et al. 2022), which could explain their relatively reduced likelihood of confidence. It was a concern that therapists had no equivalence of a training year in practice postqualification with an educational supervisor (which the dentists do in the United Kingdom) until recently. A training program has been introduced, but satisfactory completion is still not a requirement for UK graduates to be registered to provide NHS dentistry, as it is for newly qualified dentists. This lack of support at an early stage may be a reason for these concerning responses.

When using Likert instruments, which ask for agreement or disagreement with a statement, there may be a tendency to agree, resulting in acquiescence bias; therefore, an attempt was made to balance broadly similar statements positively and negatively to minimize this. Confidence in placing different restorations in different situations may be interpreted as confidence in the material or in the clinician’s ability, which could lead to response bias. It was felt that although more questions could be asked to more accurately ascertain this, the facets of confidence were interlinked and repeating similar questions risked overburdening respondents for minimal additional insight and risking potential respondent fatigue bias (Egleston et al. 2011).

Limitations around sampling, survey design, and response rates have been further discussed elsewhere (REF paper 1).

Publicly funded restoration provision predominates in the United Kingdom, with amalgam the most commonly used posterior material. This limits the generalizability of the findings, although it could be comparable to other, primarily developing, countries where amalgam use is still high (Mumtaz et al. 2010; World Health Organization 2011). Data pertaining to private dentists could potentially be generalized to other countries where this is the main mode of health care provision and amalgam use is still permitted.

## Conclusion

This survey has shown that primary care dentists and therapists in the United Kingdom have some major personal and patient-centered concerns over the phase-down of amalgam. Many lack confidence with the alternative, composite, when restoring posterior teeth in difficult situations, whereas confidence in placing amalgam in similar situations is much higher. They also have limited knowledge of the details of the phase-down. There is a need for more effective education of clinicians, a greater understanding of patients’ values, and policy changes to ensure the success of any phase-down and potential phase-out of amalgam.

## Author Contributions

O. Bailey, contributed to conception, design, data analysis, and interpretation, drafted and critically revised the manuscript; C.R. Vernazza, S. Stone, L. Ternent, contributed to conception, design, data analysis, and interpretation, critically revised the manuscript; A.-G. Roche, contributed to design and data acquisition, critically revised the manuscript; C. Lynch, contributed to design, critically revised the manuscript. All authors gave final approval and agree to be accountable for all aspects of the work.

## Supplemental Material

sj-pdf-1-jct-10.1177_2380084420954766 – Supplemental material for Amalgam Phase-Down Part 2: UK-Based Knowledge, Opinions, and Confidence in the Alternatives Click here for additional data file. Supplemental material, sj-pdf-1-jct-10.1177_2380084420954766 for Amalgam Phase-Down Part 2: UK-Based Knowledge, Opinions, and Confidence in the Alternatives by O. Bailey, C.R. Vernazza, S. Stone, L. Ternent, A.-G. Roche and C. Lynch in JDR Clinical & Translational Research
