# Supplementary material for: Amalgam Phase-Down Part 2: UK-Based Knowledge, Opinions, and Confidence in the Alternatives
Source: JDR Clin Trans Res. 2020 Dec 10;7(1):50–60. doi: 10.1177/2380084420954766 (PMC8674793; doi:10.1177/2380084420954766)
Supplement: sj-pdf-1-jct-10.1177_2380084420954766 – Supplemental material for Amalgam Phase-Down Part 2: UK-Based Knowledge, Opinions, and Confidence in the Alternatives [file sj-pdf-1-jct-10.1177_2380084420954766.pdf]

*Supplemental Table 1: Training received in direct posterior composite placement*

| **Training in posterior composites** | **Yes (%)** | **No (%)** | **Unsure (%)** |
|---|---|---|---|
| Postgraduate course (n=1512) | 88 | 10 | 2 |
| Undergraduate didactic (n=1511) | 63 | 30 | 7 |
| Undergraduate clinical (n=1507) | 58 | 36 | 7 |

*Supplemental Table 2: Knowledge of the phase-down and proposed phase-out of amalgam*

| **Knowledge of amalgam phase-down: Patient groups to avoid amalgam placement** | **% correct** | |
|---|---|---|
| | **Dentist (n=1416)** | **Therapist (n=73)** |
| Children (either "children mentioned or age implying children") | 95 | 96 |
| Correct age (i.e. 15 and under) | 58 | 56 |
| Deciduous/Primary teeth | 5 | 4 |
| Pregnancy | 87 | 81 |
| Breastfeeding | 47 | 41 |
| All correct apart from deciduous teeth | 27 | 25 |
| All correct | 3 | 3 |
